# Pattern of skin disease in Ethiopian HIV‐infected patients on combination antiretroviral therapy: A cross‐sectional study in a dermatology referral hospital

**DOI:** 10.1002/ski2.28

**Published:** 2021-05-03

**Authors:** F. Shikur, H. Yeung, W. Amogne, R. Weller

**Affiliations:** ^1^ Department of Dermatovenereology School of Medicine, Addis Ababa University Addis Ababa Ethiopia; ^2^ Department of Dermatology Emory University School of Medicine Atlanta Georgia USA; ^3^ Department of Internal Medicine School of Medicine, Addis Ababa University Addis Ababa Ethiopia; ^4^ Division of Medical and Radiological Sciences University of Edinburgh Edinburgh UK

## Abstract

**Background:**

More than 90% of human immunodeficiency virus (HIV)‐infected patients will develop at least one type of skin disorder during the course of the disease. The prevalence and severity of skin disease commonly seen in HIV‐infected patients has decreased in the era of combination antiretroviral therapy (cART). Few studies in Ethiopia have shown the magnitude of skin problems among adult patients on cART. The aim of this study is to describe the pattern of skin disease among adult patients who are on cART.

**Methods:**

Cross‐sectional observational study at ALERT Hospital from April 2018 to November 2018. Patterns of clinically diagnosed skin diseases were summarized descriptively.

**Result:**

A total of 572 patients were evaluated. In total, 412 (72%) were female and the mean age of study participants was 40 (*SD* = 10.4). The median CD4 count at the time of diagnosis and start of cART were 178 (R 5‐2000) and 168 cells/μl (R 5‐1327), respectively. The mean duration of cART was 8 (*SD* = 3) years. 89.3% of patients were on first line and 7% on second line of cART regimen. Noninfectious inflammatory skin disorders (40.9%) were the most common concomitant diagnosis followed by infectious diseases (34.9%), infestation (7.7%), pigmentary disorders (6.3%) and cutaneous drug eruption (0.7%), respectively. Among the inflammatory skin disorders, 56.5% presented with eczema. One patient had Kaposi sarcoma.

**Conclusion:**

Noninfectious inflammatory skin disorders are the most common concomitant skin disease in HIV‐infected patients, with eczema being most prevalent. Infectious skin diseases were also common presentations. In our study, AIDS‐defining skin conditions were rare.

## Graphical abstract

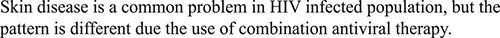

Skin disease is a common problem in HIV infected population, but the pattern is different due the use of combination antiviral therapy.

1

**What is already known about this topic?**

- Combination antiretroviral therapy has dramatically changed the natural course of HIV infection in Ethiopia.
- Skin disease is common in HIV infection in Ethiopia.
- Little is known about patterns of skin disease in cART‐treated Ethiopians with HIV.

**What does this study add?**

- Skin disease in cART‐treated HIV patient mirrors that of the non‐HIV infected population.
- AIDS defining skin disease is uncommon in cART‐treated Ethiopian patients.

## 2 INTRODUCTION

Cutaneous diseases are among the first recognized manifestations of human immunodeficiency virus (HIV) infection. Some conditions such as Kaposi's sarcoma and eosinophilic folliculitis are specific indicators of HIV, while other cutaneous diseases, while not being unique to patients living with HIV, may show more severe presentations, present atypically or be less responsive to treatment than in HIV‐negative patients.^1^, ^2^ Given the relative ease of examination of the skin, and with most skin diseases being amenable to diagnosis by inspection alone, evaluation of skin remains an important tool in the diagnosis and follow up of HIV infection.^3^ This is particularly important in resource poor countries such as Ethiopia. More than 90% of HIV‐infected patients will develop at least one type of skin disorder during the course of their HIV infection^1^ and these can reflect the underlying immune status.

The number of people dying from AIDS‐related diseases globally fell from a peak of 2.3 million in 2005 to an estimated 0.7 million in 2019.^4^ This encouragingly steep fall is a function of reduced HIV prevalence and increased access to combination antiretroviral therapy (cART). International guidelines have recognized that early treatment of HIV infection improves outcomes.^5^ Access to antiretroviral therapy has improved in the last decade for patients living with HIV in low‐ and middle‐income countries with the number of patients receiving antiretroviral therapy increasing more than 30‐fold (from 300 000 in 2002 to 9.7 million in 2012), and a resulting increase in life expectancy.^4^, ^6^ In Ethiopia, the annual number of new HIV infections similarly shows a declining trend. Incident HIV infection in adults has fallen from an estimated 13 394 in 2016 to 11 613 in 2019. Around 415 580 adults and 21 385 children were on cART and treatment coverage for adults has now reached 75% and 34% for children.^7^

The encouraging fall in HIV prevalence and mortality means that there has been a decrease in the prevalence and severity of infectious, inflammatory and neoplastic skin diseases that were commonly seen with HIV infection in the pre‐cART era. Nevertheless, we have observed that skin diseases are still commonly seen in our patients in Ethiopia receiving cART. This may be due to poor HIV control, adverse effects of cART drugs, immune reconstitution inflammatory syndrome or increased life expectancy.^8^ As few studies have examined the mucocutaneous manifestation of HIV infection outside Western countries and none has examined the pattern of skin disease in Ethiopia after the introduction of cART, we here describe the pattern of skin disease in adult patients living with HIV receiving cART at our dermatology clinic.

## 3 METHODS

We performed a cross‐sectional observational study of skin diseases in patients living with HIV and receiving cART who were seen at ALERT hospital. ALERT (All Africa Leprosy Rehabilitation and Training centre) is located in Addis Ababa, Ethiopia, and serves as the main referral hospital and training centre for skin disease for the whole of Ethiopia. Over 6000 HIV‐infected patients on cART are registered and followed up at the outpatient HIV clinic at ALERT. Between April 2018 and November 2018, adult patients in whom skin disease was noted by the HIV clinic medical staff, or who self‐identified with skin disease, were referred to the dermatology clinic. Patients had to be over the age of 18, with a diagnosis of HIV and have been on cART for at least 6 months. A complete examination of the skin, hair, nails and mucous membrane was conducted on all referred patients by a trained dermatologist or senior dermatology resident at ALERT. In most cases, a clinical diagnosis of skin disease was made. Where available, other HIV‐related parameters such as CD4 count and viral load were collected. In cases of diagnostic uncertainty, or where clinically indicated, relevant investigations, such as biopsy with a histopathological examination, mycology and microbiology were performed. Skin disorders were classified as: infection, infestation, noninfectious inflammatory condition, pigmentary abnormalities, cutaneous drug eruption and miscellaneous skin condition to allow comparison with previous surveys of Ethiopian skin disease.^9^ Data were compiled and simple summary statistics performed using SPSS version 23. Ethical approval was obtained from the Armauer Hansen Research Institute/ALERT Ethics Review Committee.

## 4 RESULTS

A total of 660 HIV‐positive patients with skin disease were referred to the dermatology clinic during the study period. Of these, complete data were available on 572 patients. Four hundred twelve patients (72%) were women and the mean age was 40 (*SD* = 10.4). All referred patients had a skin condition diagnosed. Four hundred ninety‐seven patients (86.9%) had one condition, 71 (12.4%) had two, and 4 (0.7%) had three conditions.

CD4 counts were available on 116 patients, although these were not measured at the time of diagnosis of skin disease, and we are thus not able to identify links between CD4 count and incident skin disease. Of these patients, median CD4 count at the time of HIV diagnosis was 178 cells/μl (range, 5–2000) and at the start of cART was 168 cells/μl (range, 5–1327), respectively. Viral load data were available on 263 patients, of whom 242 had no detectable viral load. The mean duration of cART in our cohort patient was 8 years. Five hundred eleven (89.3%) patients were on first‐line cART regimen and 41 (7.2%) on second‐line cART. Twenty (3.5%) further patients were on alternative regimen due to treatment failure or side effects (Table 1). In around 96% of patients’ diagnosis was made clinically. The most common additional investigation done was KOH exam for fungal infection followed by biopsy and skin slit smear test. A total of 680 mucocutaneous diagnoses were observed during the course of the study (Table 2). Noninfectious inflammatory skin conditions were the most common diagnosis, seen in 40.9% of all cases, of which eczemas (23.1%) were the most common diagnosis. Infectious skin disease was observed in 34.8% cases, with dermatophytosis (19%) being the most common diagnosis (Table 3). Of the 129 patients with a fungal infection, 65 had viral load results and 11 of these had a detectable viral load, comprising six with tinea corporis, three with onychomycosis and two with tinea facie. The pattern of fungal disease thus appeared similar in those with and without a detectable viral load. Infestation, pigmentary abnormalities, cutaneous drug eruption and miscellaneous conditions were seen in 7.7%, 6.3%, 0.7% and 9.5% of cases, respectively (Table 2). Only one patient had Kaposi's sarcoma. There was no correlation between different patterns of skin disease identified when patients were stratified by CD4 count.

**TABLE 1:** Demographic characteristics (*n* = 572)

Variables	
Age (mean, 1*SD*)	40 (10.4)
Sex (M: F)	1:3
ART duration in years (mean, *SD*)	8, 3
Mean CD4 count	
At diagnosis of HIV (range)	178 (5–2000)
At start of cART (range)	168 (5–1327)
Viral load (number, %)	
Not detected	217 (38%)
Detected	46 (8%)
Missing	309 (54%)
cART regimen (number, %)	
First line	511 (89.5%)
Second line	41 (7%)
Other	20 (3.5%)
Other medical history (number, %)	371 (64.6%)
Tuberculosis	140 (24.3%)
Hypertension	15 (2.6%)
Diabetes	4 (0.7%)
Other	212 (36.9%)

**TABLE 2:** Dermatologic disease category

Disorder	Number	Percent
Noninfectious inflammatory	278	40.9%
Infectious	237	34.9%
Infestation	52	7.7%
Pigmentary abnormalities	43	6.3%
Cutaneous drug eruption	5	0.7%
Miscellaneous	65	9.5%
Total = 680 (number of diagnosis)		

**TABLE 3:** Prevalence of skin manifestation

Noninfectious disorders		Infectious disorders		
Disorder	Number, %	Disorder		Number, %
**Eczema**	157, 23.1	**Fungal**		129,19
Seborrheic eczema	64	Tinea corporis		33
Allergic contact eczema	23	Onychomycosis		23
Atopic eczema	21	Tinea pedis		22
Discoid eczema	19	Tinea facie		18
Others	30	Vaginal candidiasis		8
**Papulosquamous**	20, 3	Oral trash		6
Lichen planus	15	Tinea capiti		5
Psoriasis	11	Others		14
Pityriasis rosea	1	**Bacterial**		43, 6.3
**Pilosebaceous**	20, 3	Furuncle		14
Rosacea	7	Bacterial folliculitis		11
Perioral dermatitis	4	Carbuncle		9
Alopecia areata	4	Leprosy		2
Acne	3	Others		7
Folliculitis decalvance	2	**Viral**		61, 8.9
**Other inflammatory**	91, 13.4	Genital wart		16
Pruritic papular eruption	46	Molluscum cotagiosum		11
Acute urticarial	15	Herpes zoster		10
Eosinophilic folliculitis	3	Common wart		9
Others	27	Plantar wart		8
**Drug eruption**	5, 0.7	Flat wart		8
Maculopapular	1	Others		3
Fixed drug eruption	1	**Protozoal**		4, 0.6
Stevens Johnson syndrome	1	Cutaneous leishmaniasis		2
Lichenoid	1	Mucocutaneous leishmaniasis		2
Urticarial	1	**Infestation**		52, 7.6%
**Pigmentary**	43, 6.3%	Scabies		52
Melasma	35			
Vitiligo	5			
Others	3			

## 5 DISCUSSION

This is the first study of prevalent skin disease in patients living with HIV infection in Ethiopia. Such studies have been carried out in other geographical regions, but the data here presented show the specific pattern of disease in the Horn of Africa which may represent both the background burden of skin disease and local factors predisposing to this pattern of disease. We did not measure the proportion of HIV‐infected patients with concomitant skin disease, but instead the pattern of skin disease in those with mucocutaneous involvement. The robust data that we have generated will allow for healthcare planning and alerts physicians to conditions they should anticipate treating.

The effectiveness of cART has meant that even in low‐ and middle‐income countries, HIV infection is a chronic condition, and the patterns of skin disease seen are more likely to represent the underlying population pattern of skin disease rather than the specific HIV defining skin conditions seen in the pre‐cART era.

We identified a relatively low number of AIDS‐defining skin diseases at ALERT during the 7 months of our study period. The most prevalent that we observed were mild to moderate seborrheic eczema in 11% (64 cases, five with a detectable viral load), and pruritic papular eruption in 8% (46 cases, six with detectable viral load). Pruritic papular eruption has been reported with a range of prevalence from 12% to 46% in Sub‐Saharan Africa^7^ and was also the most prevalent inflammatory manifestation of HIV infection at 16% in an Indian study.^10^ It thus remains relatively common despite cART. Five drug rashes were seen, which is an unusually low number but probably reflects the fact that patients were seen in a routine clinic, and patients with an acute drug rash would generally have been referred urgently to their local hospital. Three drug rashes were due to antibiotics (azithromycin, norfloxacin and ciprofloxacin) and in the others we were unable to identify the offending drug. This is lower than 3.9% reported in central Africa.^11^ No drug eruptions specifically due to cART were seen, probably because such reactions generally occur in the first few weeks after initiation of therapy, and our cohort were well established on these drugs. Eosinophilic folliculitis is usually seen with severe immunosuppression but was only diagnosed in three of our patients (one with a detectable viral load) which may be an indicator of the success of cART in controlling immunosuppression in this Ethiopian setting. A single case of Kaposi sarcoma (KS) was diagnosed. In America and Europe, KS is particularly prevalent in Men who have Sex with Men (MSM)^12^ and the very low case rate we saw may reflect the different epidemiology for HIV infection in Africa, as well as good disease control with cART to which KS responds.

Noninfectious inflammatory conditions as a group were the most common skin disorders (40.9%), particularly eczemas (seborrheic dermatitis 11%, and eczemas other than seborrheic dermatitis 16%). This pattern of skin disease is not dissimilar to that of non‐HIV‐infected Ethiopian skin patients. In the Dermatology Department of Black Lion Teaching Hospital in Addis Ababa, eczemas make up 20%–25% of all adult outpatient consultations, and skin infections and infestations another 25%.^9^ The high prevalence of inflammatory skin disease in our cART‐treated HIV‐infected patients thus reflects the general Ethiopian population's pattern of skin disease. The American experience of patients on cART is that folliculitis and prurigo nodularis are the most prevalent skin conditions^13^; we suspect this may be due to a different background pattern of skin disease. In Thailand, 61% of patients had noninfectious inflammatory conditions,^14^ which is thus much closer to the Ethiopian than American pattern of disease. The high prevalence of women in our cohort (412, 72%) was also reported by Salmani et al. in Nigeria and Glynn et al. in Kenya.^1^ Photosensitivity is common in cART‐treated patients in temperate countries affecting 5%–8% of patients.^15^ Ethiopia is on the equator and mostly at an altitude of around 2500 m, so has an extremely high UV insolation. In the non‐HIV‐infected population, photodermatoses are common, yet the only photosensitivity we detected was actinic lichen planus in 2.2% of our cohort. This surprising finding may be due to patients on cART being cautious of sun exposure. The incidence of psoriasis is not affected in HIV‐infected population but the presentation can be aggressive.^8^ Despite this, in our study, only mild to moderate chronic plaque type of psoriasis was seen in 1.2% of cases.

Infectious cutaneous disorders were the second most common presentation in our study. Fungal infections were the most common (129, 19%), similar to a Chinese study that suggested that fungal infection might be considered as a marker of disease progression.^16^ Tinea corporis was most common as in a Nigerian study.^1^

Viral infections were the second most common infectious conditions with genital warts being most prevalent, followed by molluscum contagiosum. Zancanaro et al.^13^ reported an increased number of molluscum contagiosum. Meys et al.^17^ also reported that there is no demonstrable effect of cART in reducing cutaneous warts. Cutaneous leishmaniasis is endemic and highly prevalent in the highland area of Ethiopia.^18^ The country has seen new outbreaks in areas previously not known to be endemic, often with coinfection by the HIV with rates reaching 5.6% of the cases.^19^ It can present with atypical presentation in patients living with HIV infection but the usual manifestation is more frequent. In our study 0.6% of cases were cutaneous leishmaniasis and we did not observe any atypical manifestations of the disease. Seven point six percent of patients had scabies in our cohort, but this is probably similar to the healthy population prevalence, as scabies was highly prevalent throughout Ethiopia at the time of data collection. No cases of crusted scabies were seen. We observed unexpectedly low pigmentary changes, with no identified melanonychia although this is reported in patients on cART. Similarly, we only identified 4 (0.7%) cases of xerosis despite other studies reporting magnitudes as high as 75%.^16^ This may represent underreporting of xerosis or real changes in prevalence due to local factors.

## 6 LIMITATION

This is a cross‐sectional study and substantial proportion of patients did not have a viral load measured; therefore, it is difficult to establish an association between CD4 count, viral load and skin diseases within this small‐scaled study. Future longitudinal studies will be required to understand the trend of skin diseases experienced by patients living with HIV as cART continues to evolve. The study is also limited to a single referral centre at ALERT, therefore we cannot generalize to other settings in Ethiopia.

## 7 CONCLUSION

In this Ethiopian referral hospital, we observed a low number of AIDS‐defining skin diseases in cART‐treated HIV‐positive patients. Inflammatory and infectious skin diseases broadly matched the background pattern of skin disease in the general population. Noninfectious inflammatory dermatoses were the most common skin manifestations. Dermatophytosis and pruritic papular eruption were commonly seen in patients with detectable viral load despite treatment with cART.

## CONFLICT OF INTERESTS

No conflict of interests have been declared.

## Data Availability

The data that support the findings of this study are available from the corresponding author upon reasonable request.
